# The mTOR inhibitor Everolimus synergizes with the PI3K inhibitor GDC0941 to enhance anti-tumor efficacy in uveal melanoma

**DOI:** 10.18632/oncotarget.8054

**Published:** 2016-03-14

**Authors:** Nabil Amirouchene-Angelozzi, Estelle Frisch-Dit-Leitz, Guillaume Carita, Ahmed Dahmani, Chloé Raymondie, Géraldine Liot, David Gentien, Fariba Némati, Didier Decaudin, Sergio Roman-Roman, Marie Schoumacher

**Affiliations:** ^1^ Translational Research Department, Institut Curie, PSL Research University, Paris, France; ^2^ Laboratory of Preclinical Investigation, Translational Research Department, Institut Curie, PSL Research University, Paris, France; ^3^ Unité Mixte de Recherche 3347, Institut Curie, PSL Research University, Orsay, France; ^4^ Genomics Platform, Translational Research Department, Institut Curie, PSL Research University, Paris, France; ^5^ Department of Medical Oncology, Institut Curie, PSL Research University, Paris, France; ^6^ Current address: Candiolo Cancer Institute - FPO, IRCCS, Candiolo, Torino, Italy

**Keywords:** uveal melanoma, preclinical models, PI3K, mTOR, apoptosis

## Abstract

Uveal melanoma (UM) is the most frequent malignant ocular tumor in adults. While the primary tumor is efficiently treated by surgery and/or radiotherapy, about one third of UM patients develop metastases, for which no effective treatment is currently available. The PKC, MAPK and PI3K/AKT/mTOR signaling cascades have been shown to be associated with tumor growth. However, none of the compounds against those pathways results in tumor regression when used as single agents. To identify more effective therapeutic strategies for UM patients, we performed a combination screen using seven targeted agents inhibiting PKC, MEK, AKT, PI3K and mTOR in a panel of ten UM cell lines, representative of the UM disease. We identified a strong synergy between the mTOR inhibitor Everolimus and the PI3K inhibitor GDC0941. This combination resulted in an increase in apoptosis in several UM cell lines compared to monotherapies and enhanced the anti-tumor effect of each single agent in two patient-derived xenografts. Furthermore, we showed that the synergism between the two drugs was associated with the relief by GDC0491 of a reactivation of AKT induced by Everolimus. Altogether, our results highlight a novel and effective combination strategy, which could be beneficial for UM patients.

## INTRODUCTION

Uveal melanoma (UM) is the most common primary intraocular malignancy in adults with an average incidence of 5 cases per million in Caucasian countries [1]. Despite improvement of diagnosis and treatment of the primary tumor, there is no effective treatment of the metastatic disease and approximately one third of patients die within one year or less following metastasis development [2–4]. More than 80% of UM have mutations in the G proteins GNAQ/GNA11, which activate the protein kinase C (PKC), MAPK and Hippo/YAP signaling pathways [5–7]. As a consequence, several preclinical studies with PKC and MEK inhibitors have been conducted over the last years [6]. However, inhibition of PKC or MEK alone is not sufficient to completely eliminate tumor cells or to reduce tumor burden in animals. Moreover, no improvement in overall survival has been demonstrated in clinical trials when compounds against these targets were used as monotherapies [8, 9]. In order to improve the outcome of UM patients, new therapeutic strategies are therefore warranted.

Preclinical models, such as cell lines and patient-derived xenografts (PDX) that accurately reproduce the molecular features of UM and display a high predictive value for clinical efficacy in patients are critically needed for the development of new treatments. We have recently described the establishment of a panel of relevant UM cell lines, in which we have shown the efficacy of RAD001 (Everolimus), a selective inhibitor of mTOR and subsequently of the PI3K/AKT/mTOR pathway [10]. However, even if a significant growth inhibition was demonstrated both *in vitro* and *in vivo*, treatment with RAD001 failed to induce apoptosis and tumor regression. Co-inhibition of the PI3K/AKT/mTOR and PKC/MAPK pathways has been suggested as a potential therapeutic approach for UM [11–13]. However, these studies reported only partial responses and were performed in a limited number of cell lines; they would thus need to be validated in additional UM models. So far, no preclinical study comparing drug combinations in a large panel of relevant UM cell lines has been conducted.

Here, we aimed to identify novel combination strategies that could overcome the low efficacy observed *in vitro* and *in vivo* with monotherapies. We performed a drug combination screen in our panel of UM cell lines using compounds targeting key effectors of the PKC, MAPK and PI3K/AKT/mTOR pathways. For the most synergistic combinations, cell cycle and apoptosis were evaluated *in vitro*. The best combination was then further investigated using molecular analyses to understand its mechanism of action and tested *in vivo* in UM PDXs.

## RESULTS

### Identification of synergistic combinations in uveal melanoma cell lines

**Figure 1:**
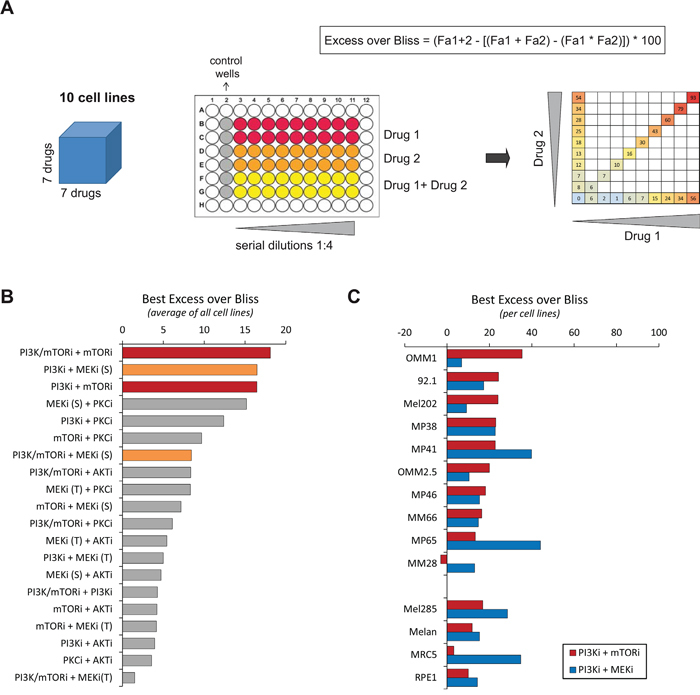
Results of the drug combination screen **A.** Scheme illustrating the screening methodology. The screen was done in ten cell lines, using seven drugs used alone or in combination *(left)*. Each drug association was tested in serial dilutions *(middle)* and partial combination matrix was obtained for each cell line and each combination *(right)*. The Excess over Bliss was calculated for each concentration. Fa: Fractional activity. **B.** Classification of all tested combinations according to their Excess over Bliss values. The highest value for each cell line was taken; the average for all cell lines was calculated and used to classify the combination activities. The drug associations with the highest Excess over Bliss values are highlighted in red and orange. **C.** Results per cell line of the chosen highly synergistic drugs. PI3Ki = GDC0941; mTORi = RAD001 (Everolimus); MEKi (S) = AZD6244 (Selumetinib). See Supplementary Table S2 for the listing of each drug.

To identify novel therapeutic approaches for UM, we performed a drug combination screen in which all possible 2×2 drug combinations between seven targeted compounds were tested across a panel of ten UM cell lines (Figure 1A; Supplementary Tables S1 and S2). Four control lines were included to assess specificity towards UM with GNAQ/11 mutations: the immortalized cells from the retina RPE1, the normal lung fibroblasts MRC5, a GNAQ/11 wt UM line Mel285 and the human normal uveal melanocytes Melan3. Compound selection was based on the main signaling cascades deregulated in UM and for which specific inhibitors are available: PKC, MAPK and PI3K/AKT/mTOR pathways (Supplementary Table S2). Each 2×2 combination was tested at multiple concentrations using a diagonal matrix in which each drug was added either as single agent or in combination (Figure 1A). All combinations were assessed for synergy based on cell proliferation and according to the Bliss independence model (Figure 1A). To classify all combinations according to their synergy strength, we calculated the average score for each combination taken into account the highest Excess over Bliss value for each cell line. Among the 20 evaluated drug associations, the top 3 synergistic ones were combinations between (1) dual PI3K/mTOR + mTOR inhibitors (PI3K/mTORi + mTORi = BEZ235 + RAD001), (2) PI3K + MEK inhibitors (PI3Ki + MEKi(S) = GDC0941 + Selumetinib/AZD6244), (3) PI3K + mTOR inhibitors (PI3Ki + mTORi = GDC0941 + RAD001) (Figure 1B).

Since the combination between PI3K and MEK inhibitors has already been described as an effective combination in UM [12], we focused on the co-inhibition of PI3K and mTOR which showed a similar synergy in our screen. Even if the combination between the dual mTOR/PI3K inhibitor BEZ235 and mTORi scored as the highest synergistic combination, we did not include BEZ235 in our follow-up studies in order to compare the effect of the association of the PI3Ki with MEKi versus mTORi. We selected GDC0941, RAD001 and AZD6244 as tool compounds to inhibit respectively PI3K, mTORC1 and MEK activities. The highest Excess over Bliss value for each cell line in PI3Ki + mTORi and PI3Ki + MEKi combinations is represented in Figure 1C. Importantly, the PI3Ki + mTORi combination showed higher scores in most of our panel of cell lines compared to PI3Ki + MEKi, with the exception of the MP41 and MP65 models. The Excess over Bliss at each drug concentration and the corresponding partial matrices are depicted in Supplementary Figures S1 and S2.

### Dual inhibition of PI3K and mTORC1 strongly induces apoptosis in the synergistic model Mel202 but not in the non-synergistic cell line MM28

Since the synergy was measured based on cell proliferation and not cell death, we first compared the phenotypes associated with the two chosen combinations by looking at cell cycle regulation and apoptosis after 72h of treatment. We selected two representative cell lines: a synergistic model Mel202 and a non-synergistic (PI3Ki + mTORi) or less synergistic (PI3Ki + MEKi) one MM28. All molecular analyses were done at a drug concentration for which most cell lines had their highest Excess over Bliss value: 2.5μM for both GDC0941 and RAD001 (Supplementary Figure S1). Full 6×6 matrices were performed in all cell lines to confirm the dose range for synergistic activity (Supplementary Figures S3 and S4).

**Figure 2:**
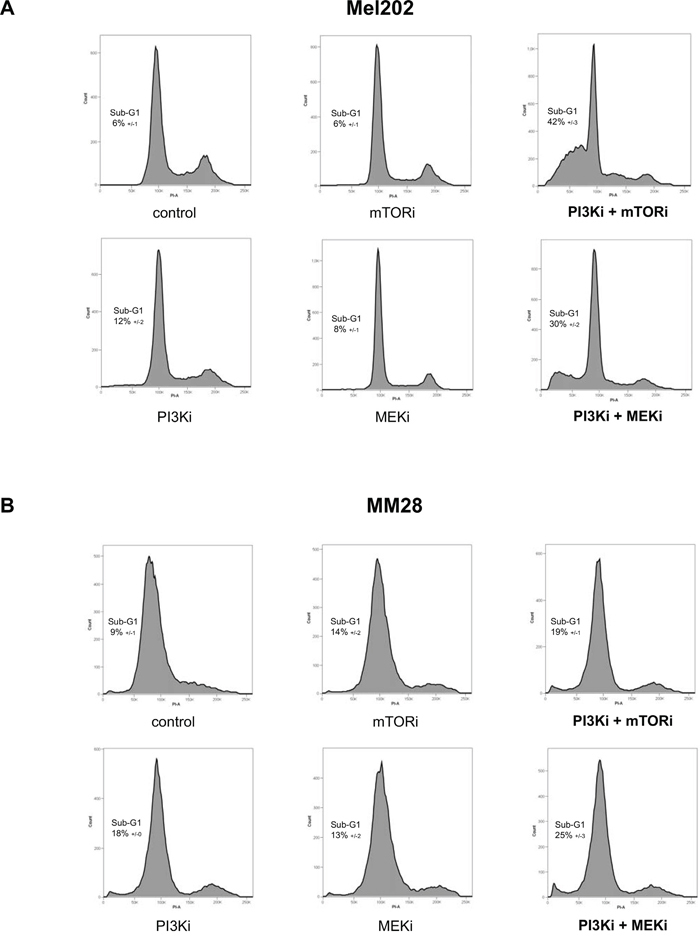
Cell cycle analyses after mTOR, PI3K and MEK inhibition alone or in combination **A.** Mel202 cell line – cell line with synergistic activities for both PI3Ki + mTORi and PI3Ki + MEKi combinations. **B.** MM28 cell line – cell line with no synergistic activity for both combinations. Results of one representative experiment are shown. The percentage of cells in Sub-G1 (apoptotic population) is represented as the mean between two independent experiments ±SD.

Cell cycle regulation was analyzed by flow cytometry (Figure 2). Treatments with mTORi, PI3Ki or MEKi as single agent did not significantly affect the cell cycle in Mel202 and MM28 cells. In Mel202 samples, a marked sub-G1 peak was observed after treatment with PI3Ki + mTORi and PI3Ki + MEKi (42 ±3% and 30 ±2% respectively compared to 6 ±1% in control) (Figure 2A). Importantly, no gain in the sub-G1 population was detected in combination treatments compared to single agents in MM28 samples (Figure 2B). The percentage of cells in each cell cycle phases and statistical analyses are represented in Supplementary Figure S5. Together, these findings suggest that the combination activity strength correlates with apoptosis induction, at least in the Mel202 and MM28 models.

**Figure 3:**
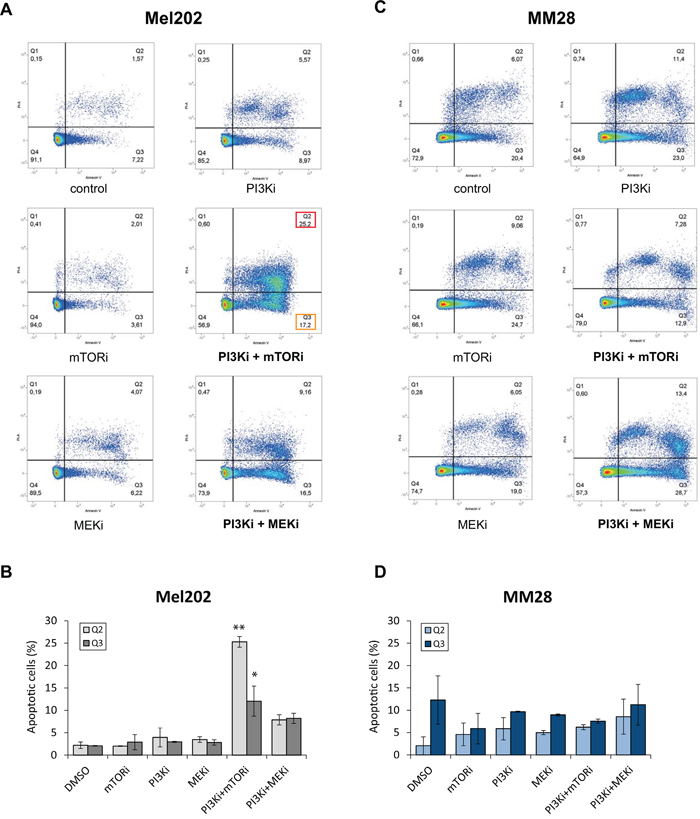
Quantification of apoptosis by Annexin V after mTOR, PI3K and MEK inhibition alone or in combination **A-B.** Mel202 cell line – cell line with synergistic activity for both PI3Ki + mTORi and PI3Ki + MEKi combinations. **C-D.** MM28 cell line – cell line with no synergistic activity for both combinations. Results of one representative experiment are shown. A and C: Results of flow cytometry analyses. Percentages of cells in each population are represented. Apoptotic populations are highlighted for the combinations: Q2 in red (late apoptotic cells) and Q3 in orange (early apoptotic cells). B and D: Quantification of all apoptotic cells (Q2 and Q3). Results of two independent experiments are combined and shown as mean ±SEM. *p<0.01, **p<0.001 by two-way ANOVA with Bonferroni correction.

To confirm this observation, we measured the proportion of apoptotic cells by AnnexinV staining. Results of one representative experiment are shown in Figure 3. In the synergistic cells Mel202, a strong and significant increase in apoptotic (Q3 = 12 ±3%; p<0.01) and late apoptotic cells (Q2 = 25 ±1%; p<0.001) was detected after treatment with PI3Ki + mTORi compared to controls (Q3 = 2 ±0.1%; Q2 = 2 ±0.7%), while single agents had no effect. No significant change was observed with the PI3Ki + MEKi combination (Figure 3A and 3B). Again, no significant difference in the apoptotic population was detected in the non-synergistic line MM28 following single agent or combination treatments (Figure 3C and 3D).

In conclusion, our results demonstrate that co-inhibition of PI3K with mTORC1 and co-inhibition of PIK3 with MEK have the strongest synergistic activity among our panel of UM cell lines. Importantly, our data further highlight that the PI3Ki + mTORi treatment induces apoptosis on contrary to the PI3Ki + MEKi combination, in at least one synergistic model.

### The reactivation of AKT by mTORC1 inhibition is removed by the combination of PI3K and mTORC1 inhibitors

**Figure 4:**
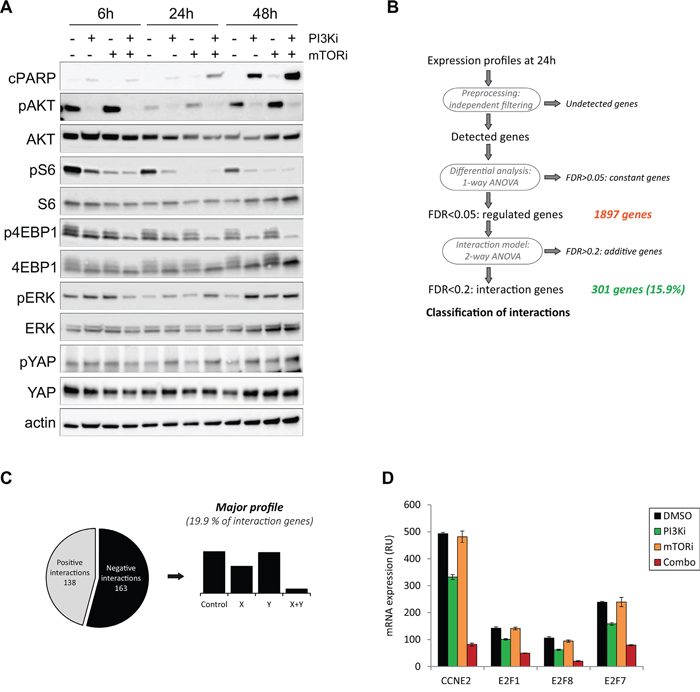
Mechanistic studies for the PI3K and mTOR inhibitor combination in the Mel202 cell line Drugs were used at 2.5μM final. **A.** Western Blot analyses for cPARP and key signaling pathways in UM. pAKT corresponds to the S473 phosphorylation site. **(B-D)** Gene expression analyses. **B.** Scheme of the method of analysis. **C.** Representation of the main pattern obtained. **D.** Examples of genes involved in cell cycle regulation and downregulated in the combination.

Having observed a strong synergy and apoptosis between PI3Ki and mTORi, we next asked what would be its mechanism of action and performed molecular analyses in the synergistic model Mel202 (Figure 4). The induction of apoptosis by PI3Ki + mTORi treatment was confirmed using an antibody against cleaved PARP (cPARP).

We first interrogated the activities of known deregulated signaling cascades in UM and compared treatments with DMSO control, PI3Ki or mTORi alone and their combination. Samples were analyzed after 6h, 24h and 48h of compound addition (Figure 4A). To verify that the PI3Ki and mTORi were indeed able to block activity of the PI3K/AKT/mTOR pathway, expression levels of phosphorylated AKT (pAKT), phosphorylated S6 (pS6) and phosphorylated 4EBP1 (p4EBP1) were analyzed. As soon as 6h after treatment, PI3Ki reduced pAKT, pS6 and p4EBP1 levels, while mTORi decreased pS6 levels only. The effects on pS6 and p4EBP1 were more pronounced at later time points. Notably, a complete loss of phosphorylation on AKT, S6 and 4EBP1 was observed only after co-inhibition of PI3K and mTORC1, indicating that inhibition of both proteins was necessary to fully block the PI3K/AKT/mTOR pathway activity as previously shown [14]. The particularly strong synergy observed between PI3Ki and mTORi led us think that additional mechanisms may explain the combination activity and enhance the anti-proliferative effect of PI3K/AKT/mTOR pathway inhibition. To test this hypothesis, we examined the activities of other signaling cascades involved in UM progression: the MAPK and Hippo/YAP pathways by looking respectively at phosphorylated ERK (pERK) and phosphorylated YAP (pYAP) (Figure 4A). No significant change in pERK levels was observed after treatment with PI3Ki or mTORi. While PI3Ki treatment slightly increased pYAP levels from 24h of treatment, no further variation was detected in the combination setting. Together, these observations show that the MAPK and Hippo/YAP pathways may not play a role in the PI3Ki + mTORi synergistic activity.

Next, we performed an unbiased study by analyzing the transcriptional response in Mel202 cells treated with PI3Ki and mTORi as single agent or in combination. To control for secondary effects arising from impaired cell proliferation, samples were collected at early time points: 6h and 24h. Given that no significant change in gene expression was detected at 6h, analyses were done with the 24h time point. Raw data were analyzed using an algorithm that detects differentially expressed genes in treatment conditions compared to DMSO-treated controls (Figure 4B) [15]. A total of 1897 genes were differentially expressed and 301 of them (15.9%) showed interaction patterns, meaning different expression in drug treatment groups compared to control (Figure 4B). Those 301 genes were then classified according to their expression profile based on a classification described in [15] (Supplementary Figure S6). We identified 138 positive (upregulated genes in the combination) and 163 negative interactions (downregulated genes in the combination), with the most enriched profile showing a negative interaction (19.9% of genes; 60 genes in total) (Figure 4C). To gain insight into this specific category, a gene set enrichment analysis was performed and identified cell cycle regulation and DNA replication as the two most enriched pathways (Supplementary Table S3). In particular, genes in the E2F family of transcription factors and Cyclin E2 belonged to this category and were strongly downregulated after combination treatment (Figure 4D). Together, this analysis confirms that cell cycle regulation is impaired after PI3Ki + mTORi treatment but fails to identify additional mechanisms that could explain the high synergistic activity between the two drugs.

Removal of feedback loops are common mechanisms of drug combination activity and often explain the synergy observed between two compounds [16]. In particular, treatment with inhibitors of mTORC1 such as RAD001 has been shown to induce a feedback loop on AKT through activation of IRS1 and mTORC2 or receptor tyrosine kinases (RTKs) [17–19]. Hence, RAD001 treatment can increase AKT activity and reactivate its downstream pathway. Following these findings, combinations of PI3K and mTORC1 inhibitors were proved to enhance the anti-proliferative effect of the single agents [14, 20]. We then questioned if such a mechanism occurred in our models. By looking at the molecular analyses in the synergistic Mel202 cells, we observed that mTORi (RAD001) treatment slightly increased pAKT compared to DMSO at all time points (Figure 4A), suggesting that the reactivation of AKT upon mTORC1 inhibition could also be valid in UM models and may be part of the mechanism for PI3Ki + mTORi combination activity.

### The induction of apoptosis by co-inhibition of PI3K and mTORC1 correlates with the removal of the reactivation of AKT in most UM cell lines

We next questioned if the observation made in the Mel202 model could be validated in the other UM cells.

**Figure 5:**
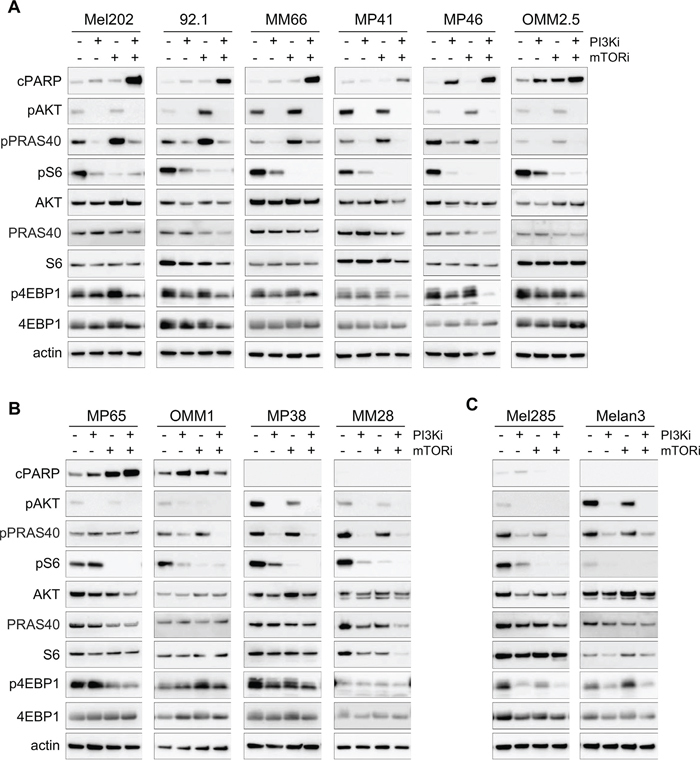
Molecular analyses for the PI3K and mTOR inhibitor combination in all cell lines Each model was analyzed after 72h of treatment for apoptosis (cPARP) and activity of the PI3K/AKT/mTOR pathway. pAKT corresponds to the S473 phosphorylation site. **A.** Cell lines with induction of apoptosis after PI3Ki + mTORi combination treatment and displaying feedback on AKT/PRAS40 after mTOR inhibition. **B.** Cell lines with no feedback on AKT/PRAS40 after mTORi (MP65) and/or no induction of apoptosis in the combination (OMM1, MP38, MM28). **C.** Control cell lines with no apoptosis and no feedback on AKT/PRAS40.

To first assess if apoptosis induction was a common phenotype associated with the PI3Ki + mTORi synergy, we evaluated the apoptotic response in our entire panel of UM cell lines. Western blot analyses were performed after 72h of treatment with PI3Ki, mTORi alone or in combination (Figure 5). We verified that the targeted pathways were inhibited by each drug. In all cells, abolition of pAKT and reduction of p4EBP1 and pS6 levels were observed after PI3Ki treatment. Treatment with mTORi only reduced pS6 but more strongly than PI3Ki. As in the Mel202 model, co-inhibition of PI3K and mTOR was necessary to fully inhibit PI3K/AKT/mTOR pathway activity (loss of phosphorylation on AKT, S6 and 4EBP1). A marked induction of cPARP was observed upon combination treatment compared to monotherapies in Mel202, 92.1, MM66, MP41, MP46, OMM2.5 and MP65 cell lines (Figures 5A and 5B left). In OMM1 cells, the PI3Ki + mTORi combination did not result in an increase of the apoptosis already induced by each single agent (Figure 5B). In the two remaining cell lines, MP38 and MM28, as well as in the control lines Mel285 and Melan3, no apoptotic effect of either single or combination treatments was observed (Figures 5B and 5C). In conclusion, these results show that co-inhibition of PI3K and mTORC1 induced or increased apoptosis in most UM cell lines (70%; 7 out of 10) compared to single treatments. The fact that not all synergistic cell lines displayed apoptosis could be explained by the measurement of the synergy strength based on cell growth rather than cell death.

We next asked if mTORi could induce the reactivation of AKT in all synergistic models and examined whether the relief of this feedback mechanism could be associated with combination activity and apoptosis induction. To measure more precisely AKT activity, samples were probed for phosphorylated PRAS40 (pPRAS40), a direct substrate of AKT. In the majority of apoptotic models, an increase in pPRAS40 after mTORi was observed, demonstrating that mTORC1 inhibition indeed induced a reactivation of AKT (Figure 5A). Interestingly, addition of PI3Ki led to complete reduction of pPRAS40 levels and thus AKT activity (Figure 5A). In the other models, the reactivation of AKT was not observed upon mTORi treatment but full inhibition of pPRAS40 and pAKT was still obtained in the combination setting (Figure 5B). Overall, the reactivation mechanism on AKT induced by mTORi happened in 85.7% of models (6 out of 7) for which the combination induced apoptosis, while it was not detected in the other models. However, the fact that PI3Ki + mTORi co-treatment fully inhibited PI3K/AKT/mTOR activity in all models independently of the induction of apoptosis indicates that other molecular mechanisms may still mediate the synergy.

### Combination of PI3K and mTORC1 inhibitors enhances *in vivo* anti-tumor activity of the corresponding monotherapies in UM patient-derived xenografts

In order to confirm *in vivo* the results observed *in vitro*, we evaluated the efficacy of the PI3Ki + mTORi combination in two PDX models: MM52 and MM66 (Supplementary Table S4) [21]. Drug tolerability and toxicity assessment were performed as preliminary experiments and doses for each compound were chosen accordingly: the PI3Ki GDC0941 and mTORi RAD001 were administrated *per os* once daily at 100mg/kg/day and 2mg/kg/day respectively. At theses doses, no body weight loss or other signs of toxicity was observed (Supplementary Figure S7A).

**Figure 6:**
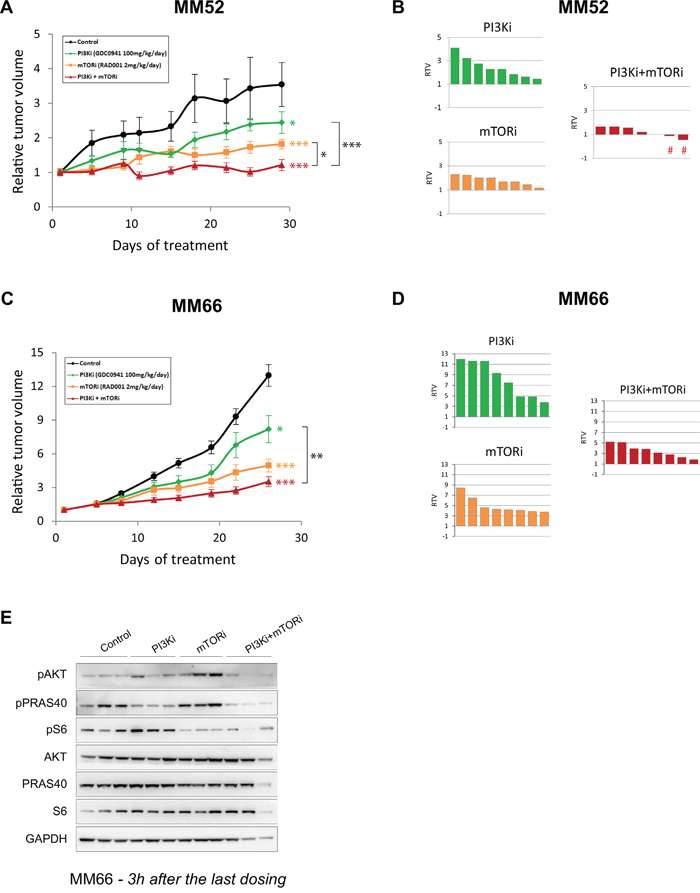
*In vivo* activity of PI3K and mTOR inhibitor combination in PDX models **A-B.** MM52 model. **C-D.** MM66 model. A and C: Relative tumor growth under treatment. Mean relative tumor volume ± SD are represented. Bilateral two-tailed Mann-Whitney tests: * p<0.05; ** p<0.005; *** p<0.0005. B and D: Relative tumor volume (RTV) per mouse for each treatment group. The signs # highlight the two mice displaying tumor shrinkage. **E.** Western Blot analyses on samples from MM66 PDX model at the end of experiment. Samples were collected and 3h after the last dosing. Three mice per group are represented.

In both PDXs, single agent treatments reduced tumor growth with a higher anti-tumor effect obtained with mTORi compared to PI3Ki. Notably, the combination treatment enhanced the anti-tumor activity of each monotherapy in the two PDX models (Figures 6A and 6C). To look more precisely at the response of each mouse in each treatment group, we represented for each mouse the relative tumor volume (RTV) which measures the tumor size at the end of experiment normalized to the one before treatment (Figures 6B and 6D). Thus, a RTV ≤ 1 indicates tumor stabilization or shrinkage. In both PDXs, the PI3Ki + mTORi combination resulted in an enhanced reduction in RTVs compared to monotherapies. Remarkably, in the MM52 model, the combination treatment led to tumor stabilization and/or tumor shrinkage in three animals (RTV ≤ 1; 3 out of 8 = 37.5%).

To confirm pathway inhibition and address whether the reactivation of AKT induced by mTORi also occurred *in vivo*, samples were collected at the end of experiment and proceeded for molecular analyses (Figure 6E and Supplementary Figure S7B). Levels of pAKT and pS6 were decreased in the combination treatment, confirming the inhibition of the PI3K/AKT/mTOR pathway. Interestingly, induction of pPRAS40 after mTORi was also observed in the MM66 model, indicating that mTORC1 inhibition could reactivate AKT *in vivo* as well (Figure 6E). The same observation was made in the MM52 model with a less pronounced increased of pPRAS40 levels probably due to the later collection time (24h compared to 3h for MM66) (Supplementary Figure S7B).

## DISCUSSION

UM remains a disease with poor outcome due to metastasis development for which no effective treatment is currently available. Drug combinations of PKCi + MEKi [11], PKCi + PI3Ki [13], MEKi + PI3Ki [12] or mTORi + MEKi [10, 22] have been tested in preclinical studies but would need to be validated in a broader spectrum of UM models in order to confirm their value for the clinics. Moreover, no simultaneous comparison of the strength of these combinations was conducted and may question which combination regimens would be more beneficial for UM patients. With the goal to discover effective therapies for UM, we performed an *in vitro* combination screen in a panel of ten UM cell lines, including seven drugs affecting the PKC, MAPK or PI3K/AKT/mTOR signaling pathways. Our data demonstrate that the PI3K inhibitor GDC0941/Pictilisib and the mTORC1 inhibitor RAD001/Everolimus synergistically induce a strong apoptotic effect *in vitro* in most UM cells and enhance tumor growth inhibition *in vivo* in two UM PDXs. Importantly, the PI3Ki + mTORi combination was more synergistic and led to higher apoptotic indexes compared to previously described combinations for UM, such as MEKi + mTOR/PI3Ki (GSK1120212/Trametinib + GSK2126458/Omipalisib [12]), mTORi + MEKi (RAD001/Everolimus + AZD6244/Selumitinib [10]; AZD8055 + AZD6244 [22]), PKCi + MEKi (AEB071/Sotrastaurin + MEK162 or PD0325901 [11]) or PKCi + PI3Ki (AEB071 + BYL719/Alpelisib [13]). Of note, compounds used in these studies were not the same as the ones tested in our screen; it would be worthwhile to evaluate those drugs in our cell line panel to confirm these observations. Especially, combinations between PI3Ki and MEKi as well as the monotherapy using mTORi are therapeutic approaches that could be worthwhile testing in clinical trials.

To understand the mechanism of the PI3Ki + mTORi combination activity, we first performed transcriptomic analyses. These studies pinpointed the inhibition of cell proliferation induced by the co-treatment but no other mechanism could be identified. Markedly, our immunoblot analyses have confirmed the reactivation of AKT induced by inhibition of the mTORC1 complex as previously described in other cancers [14, 18–20] and in the UM cell line 92.1 [23]. Several mechanisms have been suggested for this feedback, such as activation of mTORC2 through IRS1 or activation of RTKs [18, 20]. In our screen, combinations of GDC0941 + RAD001 and BEZ235 + RAD001 resulted in the highest average Excess over Bliss values. GDC0941 targets selectively p110 α/δ subunits of PI3K. BEZ235 is a dual ATP-competitive PI3K and mTOR targeting p110 α/γ/δ/β subunits and mTOR; it can therefore inhibit both mTORC1 and mTORC2 complexes. The fact that BEZ235 and GDC0941 had similar synergistic effects when combined with RAD001 suggests that the synergy derives from the outcome of either GDC0941 or BEZ235 on PI3K α/δ subunits and that mTORC2 inhibition does not contribute to the synergy. The reactivation of AKT by mTORC1 inhibition could thus be linked to RTK activation; a hypothesis that needs to be further investigated. Interestingly, we observed a correlation between the removal of the mTORi-induced AKT reactivation and the induction of apoptosis by PI3Ki + mTORi treatment. Even if both apoptotic and non-apoptotic cells presented full inhibition of PI3K/AKT/mTOR pathway after combination treatment, this observation suggests that PRAS40 phosphorylation could be used as a predictive biomarker for the response to PI3Ki + mTORi combination.

Recently, it has been shown that resistance to PI3K inhibition correlates with high levels of pS6 and thus, a high activity of the mTORC1 complex [14]. Association of PI3K with mTOR or AKT inhibitors could alleviate this resistance by enhancing PI3K/AKT/mTOR pathway inhibition. Interestingly, combination of PI3K and CDK4/6 inhibitors could remove resistance probably due to the targeting of the more downstream effector of mTORC1, Cyclin D1. In our system, we could not detect a difference in baseline pS6 levels between cell lines. According to the results of this study, it would be interesting to measure Cyclin D1 expression in our models and correlate those with the level of apoptosis induction after PI3Ki + mTORi treatment.

Finally, our panel of ten cell lines is representative of UM in terms of somatic mutations. In addition, half of them were derived from primary tumors and the other half from metastases. Even if the number of cell lines could not allow performing statistical analysis with stratification for the different variables, no correlation could be observed between response to the different combinations, mutational status or tumor origin. Interestingly, the PI3Ki + mTORi combination showed antagonism in the MM28 model. Although their slow cycling time might contribute to this phenotype, the other slow cycling models MP38, MP46 and MP65 displayed a completely different behavior, suggesting that proliferation rates cannot explain these results. A comparative study by RNA or proteomic analyses between the MM28 cells and the others could be very informative to understand the peculiarity of this model. In a similar note, we confirmed the effectiveness of PI3K and mTORC1 co-inhibition *in vivo* in two PDX models. However, the degree of anti-tumor activity was model-dependent, indicating that tumors will respond differently depending on their biological features (epigenetics, tumor microenvironment or others). It will be necessary to test additional PDXs to reflect in more details the heterogeneity of response that could be observed in patients. Evaluating more models may also lead to identification of better biomarkers to help patient stratification.

In conclusion, our work has identified the association of PI3Ki + mTORi as an effective combination in a broad spectrum of UM models and provide evidence that it could be a valuable therapeutic approach to test in clinical trials for GNAQ/11 mutated UM patients.

## MATERIALS AND METHODS

### Cell culture

MP38, MP41, MP46, MP65, MM28 and MM66 lines were established in our laboratory [10]. 92.1, Mel202 and MRC5, RPE1 cells were purchased respectively from The European Searchable Tumour Line Database (Tubingen University, Germany) and ATCC. OMM1, OMM2.5, Mel285 and Mel290 cells were kindly provided by P.A. Van Der Velden (Leiden University, The Netherlands). Cells were cultured in RPMI-1640 supplemented with 10% FBS (92.1, Mel202, OMM1, OMM2.5, Mel285, MRC5, RPE1) or 20% FBS (MP38, MP41, MP46, MP65, MM28, MM66), complemented with Penicillin at 100U/ml and Streptomycin 100μg/ml (Life Technologies). The primary culture of normal melanocytes Melan3 was isolated from a human choroid by G. Liot (Institut Curie, France) and cultured in Ham/F12 medium supplemented with 10% FBS, Penicillin/Streptavidin, FGF2 at 10ng/ml, IBMX at 0.1mM and cholera toxin at 10ng/ml. IBMX and cholera toxin were removed from the medium during drug testing to avoid interference with PKC activity. Melanocytic origin and absence of *GNAQ/11* mutation in Melan3 were validated by sequencing. All cells were Mycoplasma free and maintained at 37°C in a humidified atmosphere with 5% CO2.

### Chemicals

The MEK inhibitors AZD6244/Selumetinib and GSK1120212/Trametinib, the PKC inhibitor AEB071/Sotrastaurin, the PI3K inhibitor GDC0941, the mTOR inhibitor RAD001/Everolimus, the dual PI3K/mTOR inhibitor BEZ235, and the AKT inhibitor KRX-0401/Perifosine were supplied by Euromedex (France), dissolved in DMSO (AZD6244, GSK1120212, AEB071, GDC0941, BEZ235) or ethanol (KRX0401) at 10mM and stored at −20°C.

### Drug combination cell viability screen

At day 0, cells were seeded in 96-well plates in duplicate at appropriate concentration. At day 2, drugs were added as single agent or combination. Serial 1:4 dilutions were prepared for each drug, resulting in 10 different concentrations (including DMSO-control at 0.2% final). The highest drug concentration for each compound was decided so that the final concentrations of the two drugs produced a comparable effect and exerted their full efficacy within the first half of dilutions. Cell viability was assessed after 5 days of treatment using the MTT assay (Sigma). Results were read using a spectrophotometer and expressed as relative percentages of metabolically active cells compared with untreated controls. Cell viability was calculated as the fraction of viable cells for a given compound concentration compared to control wells. The experiments were repeated until at least an independent triplicate for each drug combination was obtained for each cell model.

### Calculation of combination activity

Combination activity was determined according to the Bliss independence definition [24]. Fractional activity (Fa) was used in all calculations (40% of cell viability equal to Fa = 0.6).

The « Excess over Bliss » was calculated as followed: (Fa1+2 - [(*Fa1* + *Fa2*) − (*Fa1* × *Fa2*)]) *100

*Fa1*, Fa2 and Fa1+2 are the fractional growth inhibitions (= fractional activity = Fa) of drug 1, drug 2 and drug1+2 at a given dose respectively. Values were calculated as the average of at least three replicates.

### Flow cytometry analyses

For cell cycle analyses, both floating and attached cells were collected, washed once with PBS, once with PBS containing 0.5% BSA and fixed with cold 70% ethanol. Then, cells were incubated in PBS containing 10μg/ml propidium iodide (PI, Invitrogen) and 200μg/ml RNaseA (Invitrogen). Samples were collected using FACScalibur (Becton Dickinson) and analyzed with CellQuest software (Becton Dickinson). DNA content was quantified by FlowJo Software (Milteny Biotec).

For apoptosis evaluation, cells were harvested after 72h of treatment. Apoptosis was measured using the AnnexinV-FLUOS staining kit (Roche) according to the manufacturer's instructions. After sequential staining by AnnexinV and PI, flow cytometry analyses were performed on a LSRII Instrument (Becton Dickinson) using the FlowJo software. The percentages of living cells (low AnnexinV and low PI), apoptotic cells (high AnnexinV and low PI) and necrotic cells (high AnnexinV and high PI) were evaluated.

Two independent experiments were performed and statistical analyses were made using two-ways ANOVA test with Bonferroni correction.

### Western blot analyses

Cells were cultured in 10cm-diameter dishes and treated with DMSO or each drug as single agent or combination for appropriate times. Western blot analyses were performed using standard procedures. Actin was used for normalization between samples. All antibodies are listed in Supplementary Materials. Signal was detected using secondary antibodies coupled with HRP (Jackson laboratory). Luminescent signal was detected using a LAS-3000 Luminescent Image analyzer.

### Gene expression profiling analysis

Mel202 cells were treated in duplicate with DMSO, 2.5μM of GDC0941, 2.5μM of RAD001 or the combination for 6 and 24 hours. Total RNA was isolated using the RNeasy Mini Kit (Qiagen). Gene expression profiling was performed using Affymetrix Human Gene 2.1ST arrays. Data were analyzed as described in [15]: profiles of differential expression between DMSO, single agents and combination were identified and classified according to their prevalence. Gene set enrichment of the most enriched profile was performed using the Molecular Signature Database (MsigDB) and David annotation.

### Patient-derived xenografts studies

Studies were performed in compliance with recommendations of the French Ethical Committee and under the supervision of authorized investigators. The experimental protocol and animal housing followed institutional guidelines as put forth by the French Ethical Committee (Agreement C75-05 -18, France) and the ethics committee of Institut Curie. Drug tolerability and toxicity were assessed in SCID mice. The maximal tolerated dose was tested in the efficacy experiments. Six week-old SCID mice were used. Tumor fragments of 30-60mm^3^ were grafted subcutaneously into the interscapular fat pad. When tumors reached a size of about 50-150mm^3^, mice were randomly assigned to control or treatment groups. Between eight to nine mice per group were included in each experiment. RAD001 was solubilized in water at 0.2mg/ml and administrated once daily *per os* at 2mg/kg/day during 5 days per week. GDC0941 was solubilized in 5% DMSO qsp water and administrated *per os* (PO) once daily at 100mg/kg/day. Treatment was done during 4 weeks and mice were then sacrificed. Tumor growth was evaluated by measuring with a caliper two perpendicular tumor diameters twice a week. Individual tumor volume and relative tumor volume (RTV) were calculated according to a standard method [21]. Tumor stability or shrinkage was defined as a RTV ≤ 1 at the end of experiments. All statistical tests were realized using a two-sided Mann-Whitney test. Results were considered statistically significant when p ≤ 0.05 (95% confidence interval).

## SUPPLEMENTARY MATERIALS FIGURES AND TABLES






