# Supplementary material for: The mTOR inhibitor Everolimus synergizes with the PI3K inhibitor GDC0941 to enhance anti-tumor efficacy in uveal melanoma
Source: Oncotarget. 2016 Mar 14;7(17):23633–46. doi: 10.18632/oncotarget.8054 (PMC5029652; doi:10.18632/oncotarget.8054)
Supplement: Supplementary file 1 [file oncotarget-07-23633-s001.pdf]

The mTOR inhibitor Everolimus synergizes with the PI3K inhibitor GDC0941 to enhance anti-tumor efficacy in uveal melanoma

SUPPLEMENTARY DATA

SUPPLEMENTARY MATERIALS

List of primary antibodies

Protein target	Supplier and catalogue number
Actin	Sigma-Aldrich
AKT	CST #9272
pAKT S473	CST #4058
S6	CST #2317
pS6 S235/236	CST #2211
ERK1/2	p44/42 MAPK CST#9102
pERK1/2	Phospho-p44/42 MAPK (T202/Y204) CST #9101
4EBP1	CST #9644
p4EBP1	CST #9459
YAP	CST #4912
pYAP	CST #4911

Supplementary Table S1: Genetic profiles of uveal melanoma cell lines screened for combinations

Cell line	MP38	MP41	MP46	MP65	MM28	MM66	92.1	Mel202	OMM1	OMM2.5	Mel285
Origin	P	P	P	P	M	M	P	P	M	M	P
GNAQ mutation	c.626 A>T	-	c.626 A>T	-	-	-	c.626 A>T	c.629 G>A	-	c.626 A>C	-
GNA11 mutation	-	c.626 A>A/T	-	c.626 A>T	c.626 A>T	c.626 A>T	-	-	c.626 A>T	-	-
BAP1 mutation	c.68-9_72del	-	-	c.1717del	c.1881 C>A	-	-	-	-	-	-
BAP1 loss	+	-	+	+	+	-	-	-	-	-	-
SF3B1 mutation	-	-	-	-	-	-	-	c.1793 C>T	-	-	-
eIF1AX mutation	-	-	-	-	-	-	c.17 G/A	-	-	-	-

P: primary tumor; M: metastasis. - = wild type/no, + = mutant/yes

Supplementary Table S2: Targeted agents used in the combination screen

Target	Compound name
PKC	AEB071 (Sotrastaurin)
MEK	GSK1120212 (Trametinib) ; AZD6244 (Selumitinib)
PI3K αβγδ	GDC0941 (Pictilisib)
mTOR	RAD001 (Everolimus)
Dual pan-PI3K/mTOR	BEZ235
AKT	KRX0401 (Perifosine)

Supplementary Table S3: Gene set enrichment analysis performed on transcriptomic data with David database

Below is a screen snap-shot of the results. More details are presented in the corresponding excel file.

See Supplementary File 1

See Supplementary File 2

Supplementary Table S4: Characteristic of the two PDX models used in the study

PDXs	Origin	Histology	*GNAQ*	*GNA11*	*BAP1*	*SF3B1*	L3
MM66	Liver metastasis	Epithelioid	wt	mut	wt	wt	N
MM52	Liver metastasis	Mixed cells	mut	wt	mut	mut	L

Abbreviations: L3: status of chromosome 3; N = normal, L = loss of heterozygocy -- Mixed cells included epithelioid and spindle cells.

A *PI3Ki + mTORi (GDC0941 + RAD001)*

PI3Ki	mTORi	92.1	Mel202	OMM1	OMM2.5	MP38	MP41	MP46	MP65	MM28	MM66	Mel285	Melan3	MRC5	RPE1
10	10	13.7	6.6	21.8	11	17.6	13.9	8.1	9.9	-8	6.4	10.5	6.8	-4.1	1.5
2.5	2.5	24.3	22.2	35.4	20	23	22.8	18.2	13.3	-6.1	15.5	11.6	1.1	-3.8	2.8
0.625	0.625	15.9	24.1	23.5	14.4	16.2	14.9	13.7	11.6	-6.7	16.4	16.9	-1	-3.1	5.2
0.156	0.156	6.7	11.1	15.7	4.1	9.8	6.7	4.7	10.9	-8.5	5.9	14.4	11.9	-2.4	3.3
0.039	0.039	0.2	4.1	9.9	5.3	6.3	7.1	-2.5	2.4	-2.8	2.2	10.9	-16.1	-0.4	5.1
0.010	0.010	1.1	3.1	5.2	1.2	3.1	-1.9	-1.8	-3.6	-8.4	2.3	7.4	2.8	-1.5	0.3
0.0024	0.0024	0.5	3.5	0.2	-1.1	4	-2.9	-3.7	4.4	-3	4.1	4.9	-2.4	3.2	3.8
0.0006	0.0006	-2.2	3.8	-2.8	-10.1	0	-2.2	-2.2	-2	-4.9	10.7	0	2	-1.7	5
0	0	1.8	2.9	-1.2	-6.6	3.8	-8.2	0.9	3.7	-3	6.3	2.3	2.7	-1.3	10
Best Excess over Bliss		***24.3***	***24.1***	***35.4***	***20***	***23***	***22.8***	***18.2***	***13.3***	***-3***	***16.4***	***16.9***	***11.9***	***3.2***	***10***

B *PI3Ki + MEKi (GDC0941 + Selumetinib)*

PI3Ki	MEKi (1)	92.1	Mel202	OMM1	OMM2.5	MP38	MP41	MP46	MP65	MM28	MM66	Mel285	Melan3	MRC5	RPE1
10	1	3.5	0.4	3.2	4	10.5	21.8	5	1.7	-4.7	2.7	18.9	5.6	0.5	-2.2
2.5	0.25	13.6	6.8	4.4	9.7	22.8	39.9	10.7	2	-3	14.8	-2.8	-7.2	-1.5	4
0.625	0.0625	10.8	9.2	5	9.5	16.7	34.4	7.9	6.7	3.3	7.7	19	-1.5	-0.1	5.1
0.156	0.0156	5.9	4.5	-2.2	7.5	8.4	21.6	6.8	-2.6	2.8	5.3	6.4	-8.1	11.5	8.9
0.039	0.0039	3.9	-7.8	-1.9	5.8	4.3	30.9	1.1	20.3	7.5	5.5	-10.6	-2.1	11.6	12.6
0.010	0.00976	9.1	0.3	-4.8	8.7	-0.8	23.1	0.7	15	3.6	-0.5	-5.8	-3.6	7.6	0.4
0.0024	0.00024	4.8	4.8	6.9	6.2	6.7	34.7	1.1	6.5	13	-1.4	13.7	-0.4	13.3	13.6
0.0006	0.00006	10.3	5.3	2.6	10.4	4.4	31.2	4.8	18.9	9.3	10.2	28.5	15.3	23	11.1
0	0	17.3	1.8	-1	3.6	7.5	21.7	15.3	44.1	8	2.8	27.1	-12.5	34.8	14.3
Best Excess over Bliss		***17.3***	***9.2***	***6.9***	***10.4***	***22.8***	***39.9***	***15.3***	***44.1***	***13***	***14.8***	***28.5***	***15.3***	***34.8***	***14.3***

Excess over Bliss = (Fa1+2 - [(Fa1 + Fa2) - (Fa1 * Fa2)]) * 100

-10 0 30

Color scale for Excess over Bliss

Supplementary Figure S1: Excess over Bliss values for the two most synergistic drug combinations. A. PI3Ki + mTORi. **B.** PI3Ki + MEKi. PI3Ki = PI3K inhibitor GDC0941; mTORi = mTORC1 inhibitor RAD001 (Everolimus); MEKi = MEK inhibitor AZD6244 (Selumitinib). All compound concentrations are shown in μM.

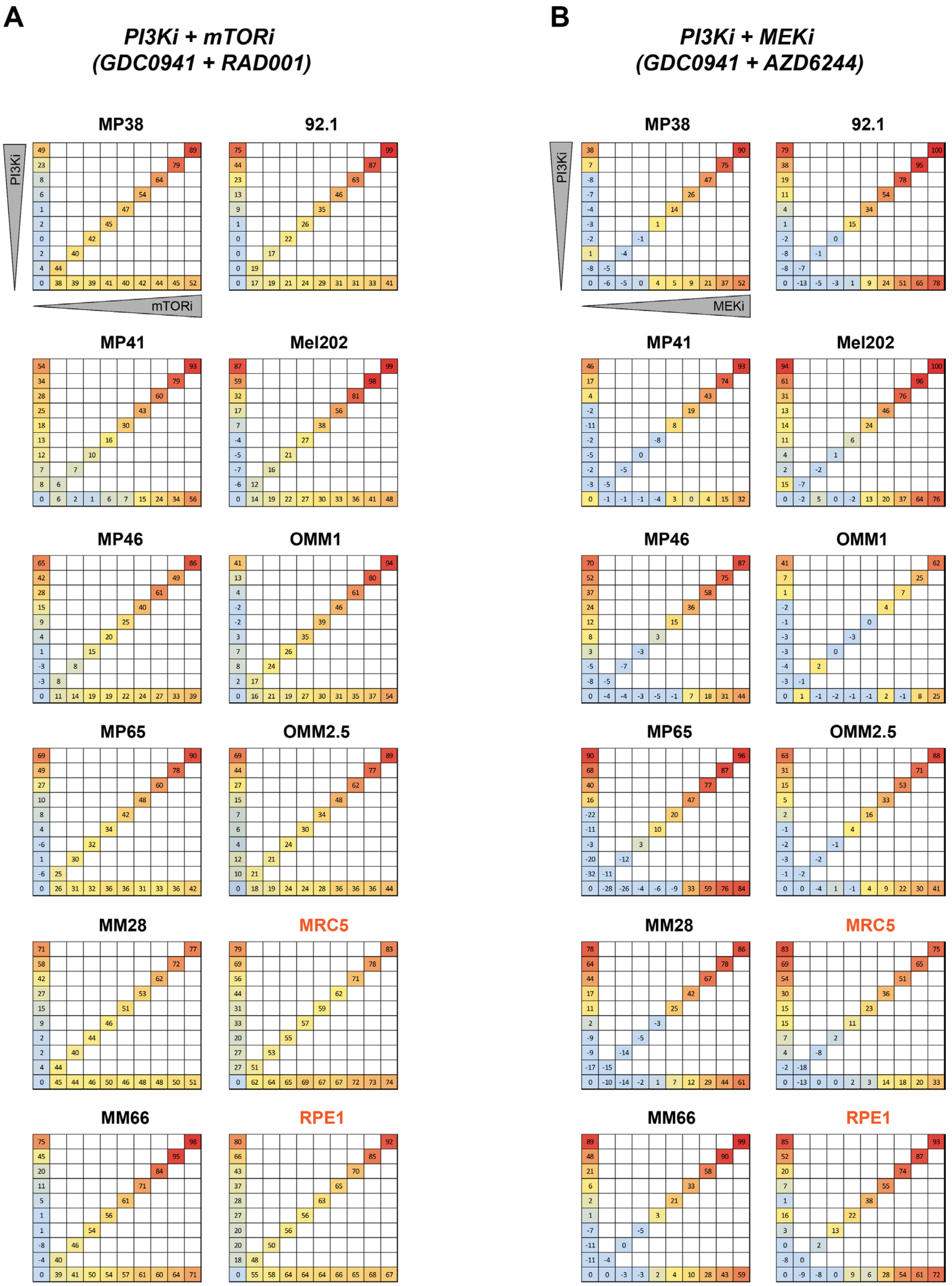

Supplementary Figure S2: Partial matrices obtained in the combination screen for the two most synergistic drug combinations. One matrix is shown per cell line and per combination. Percentages of growth inhibition compared to DMSO-treated control are shown. A color code from blue to red is used to highlight the level of growth inhibition. Names of control lines are written in orange. **A.** PI3Ki + mTORi. **B.** PI3Ki + MEKi.

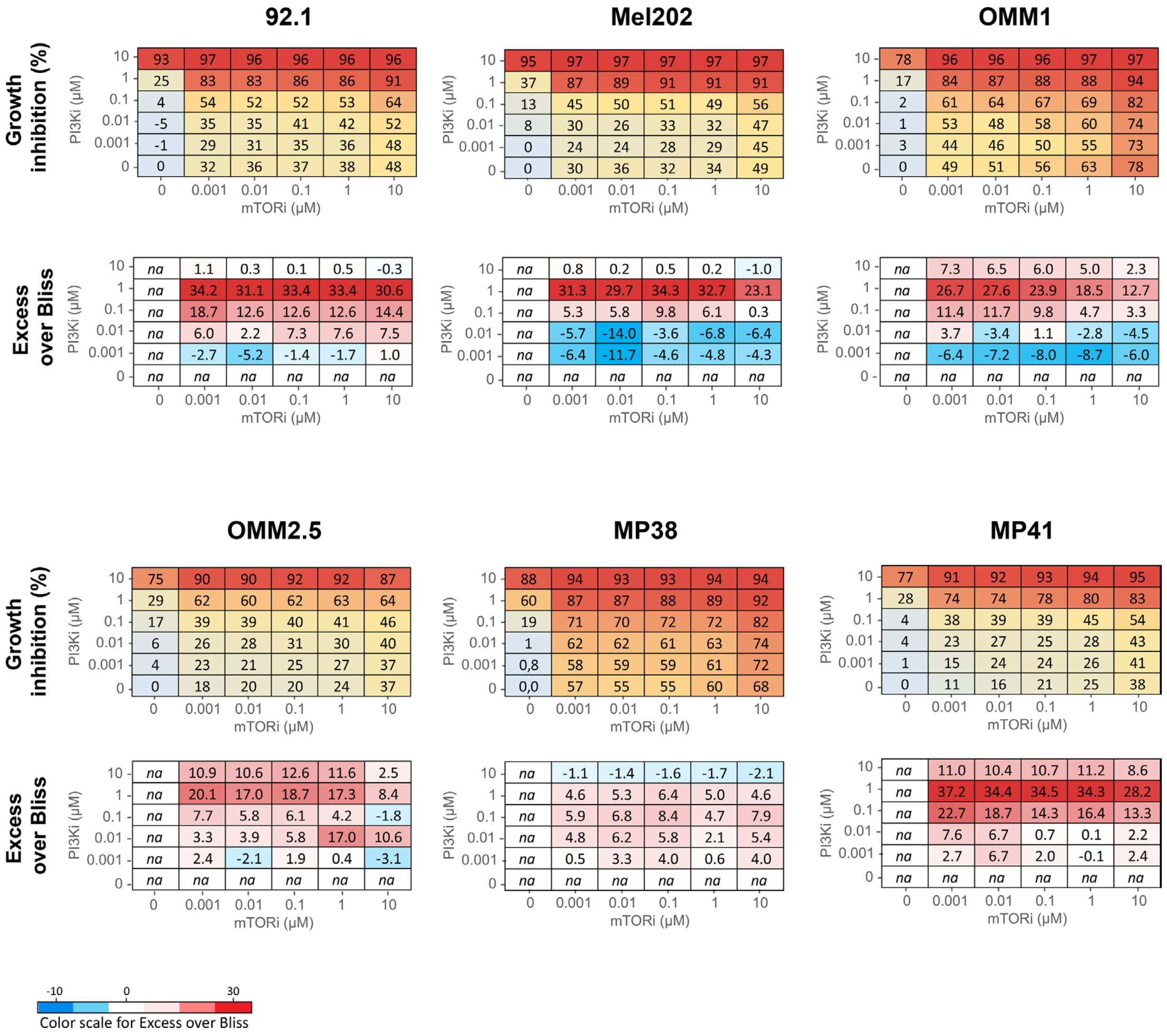

Supplementary Figure S3: Full dose matrices for the PI3Ki + mTORi combination. For each cell line, the results of growth inhibition (percentage compared to DMSO-control cells) and the Excess over Bliss values for every tested dose are shown. A color code highlights strong Excess over Bliss values (i.e. synergy) in red.

MP46

Growth inhibition (%)

PI3Ki (µM) \ mTORi (µM)	0	0.001	0.01	0.1	1	10
10	75	82	82	84	85	83
1	45	68	72	76	79	80
0.1	21	45	50	55	57	62
0.01	5	31	37	39	45	48
0.001	-7	22	26	37	37	46
0	0	24	30	38	42	47

Excess over Bliss

PI3Ki (µM) \ mTORi (µM)	0	0.001	0.01	0.1	1	10
10	*na*	1.0	0.1	0.1	-0.4	-3.6
1	*na*	10.3	10.9	9.9	11.5	8.5
0.1	*na*	4.3	4.9	3.9	3.3	3.4
0.01	*na*	3.0	3.1	-2.2	0.2	-2.0
0.001	*na*	2.4	0.6	3.1	-0.7	1.8
0	*na*	*na*	*na*	*na*	*na*	*na*

MP65

Growth inhibition (%)

PI3Ki (µM) \ mTORi (µM)	0	0.001	0.01	0.1	1	10
10	79	94	93	94	94	94
1	22	80	81	82	82	83
0.1	-2	48	50	53	49	51
0.01	0	39	35	37	37	35
0.001	6	32	35	36	38	42
0	0	33	32	34	33	38

Excess over Bliss

PI3Ki (µM) \ mTORi (µM)	0	0.001	0.01	0.1	1	10
10	*na*	8.3	7.1	7.4	7.9	6.4
1	*na*	32.1	33.8	33.3	33.6	31.1
0.1	*na*	16.3	19.5	19.3	17.2	13.2
0.01	*na*	5.9	3.6	2.5	3.6	-3.5
0.001	*na*	-4.4	-0.5	-1.7	0.7	0.4
0	*na*	*na*	*na*	*na*	*na*	*na*

MM28

Growth inhibition (%)

PI3Ki (µM) \ mTORi (µM)	0	0.001	0.01	0.1	1	10
10	88	94	93	93	94	94
1	60	87	87	88	89	92
0.1	19	71	70	72	72	82
0.01	1	62	62	61	63	74
0.001	1	58	59	59	61	72
0	0	57	55	55	60	68

Excess over Bliss

PI3Ki (µM) \ mTORi (µM)	0	0.001	0.01	0.1	1	10
10	*na*	-1.1	-1.4	-1.6	-1.7	-2.1
1	*na*	4.6	5.3	6.4	5.0	4.6
0.1	*na*	5.9	6.8	8.4	4.7	7.9
0.01	*na*	4.8	6.2	5.8	2.1	5.4
0.001	*na*	0.5	3.3	4.0	0.6	4.0
0	*na*	*na*	*na*	*na*	*na*	*na*

MM66

Growth inhibition (%)

PI3Ki (µM) \ mTORi (µM)	0	0.001	0.01	0.1	1	10
10	92	95	94	95	95	95
1	43	91	92	93	93	94
0.1	9	75	77	81	84	88
0.01	2	65	69	71	76	84
0.001	2	65	66	69	73	81
0	0	59	63	66	70	78

Excess over Bliss

PI3Ki (µM) \ mTORi (µM)	0	0.001	0.01	0.1	1	10
10	*na*	-1.9	-3.1	-2.5	-2.8	-3.4
1	*na*	14.4	13.3	12.1	10.0	7.2
0.1	*na*	11.6	11.2	11.7	11.1	8.1
0.01	*na*	5.1	5.1	4.0	4.8	5.5
0.001	*na*	4.1	2.4	2.6	2.5	3.2
0	*na*	*na*	*na*	*na*	*na*	*na*

Mel285

Growth inhibition (%)

PI3Ki (µM) \ mTORi (µM)	0	0.001	0.01	0.1	1	10
10	69	71	72	73	73	74
1	40	65	64	67	68	64
0.1	18	49	49	51	51	53
0.01	5	41	43	44	45	50
0.001	1	38	39	41	42	49
0	0	36	38	39	39	35

Excess over Bliss

PI3Ki (µM) \ mTORi (µM)	0	0.001	0.01	0.1	1	10
10	*na*	-9.4	-8.3	-8.3	-8.5	-6.3
1	*na*	2.9	1.6	3.7	3.8	-0.7
0.1	*na*	2.1	0.4	1.1	1.1	0.5
0.01	*na*	1.6	2.5	2.3	2.6	-0.2
0.001	*na*	1.0	0.4	1.5	2.2	0.8
0	*na*	*na*	*na*	*na*	*na*	*na*

Melan3

Growth inhibition (%)

PI3Ki (µM) \ mTORi (µM)	0	0.001	0.01	0.1	1	10
10	58	55	54	57	55	65
1	32	44	38	43	45	47
0.1	14	23	26	31	26	35
0.01	6	18	25	22	26	33
0.001	6	18	20	23	18	31
0	0	16	6	23	24	35

Excess over Bliss

PI3Ki (µM) \ mTORi (µM)	0	0.001	0.01	0.1	1	10
10	*na*	-9.8	-6.2	-10.6	-13.4	-8.3
1	*na*	0.9	2.3	-4.8	-3.1	-8.9
0.1	*na*	-4.8	7.5	-2.2	-8.5	-8.8
0.01	*na*	-3.0	13.5	-5.8	-3.2	-5.7
0.001	*na*	-3.4	8.5	-4.3	-11.1	-8.3
0	*na*	*na*	*na*	*na*	*na*	*na*

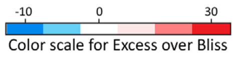

Supplementary Figure S4: Full dose matrices for the PI3Ki + mTORi combination – continued.

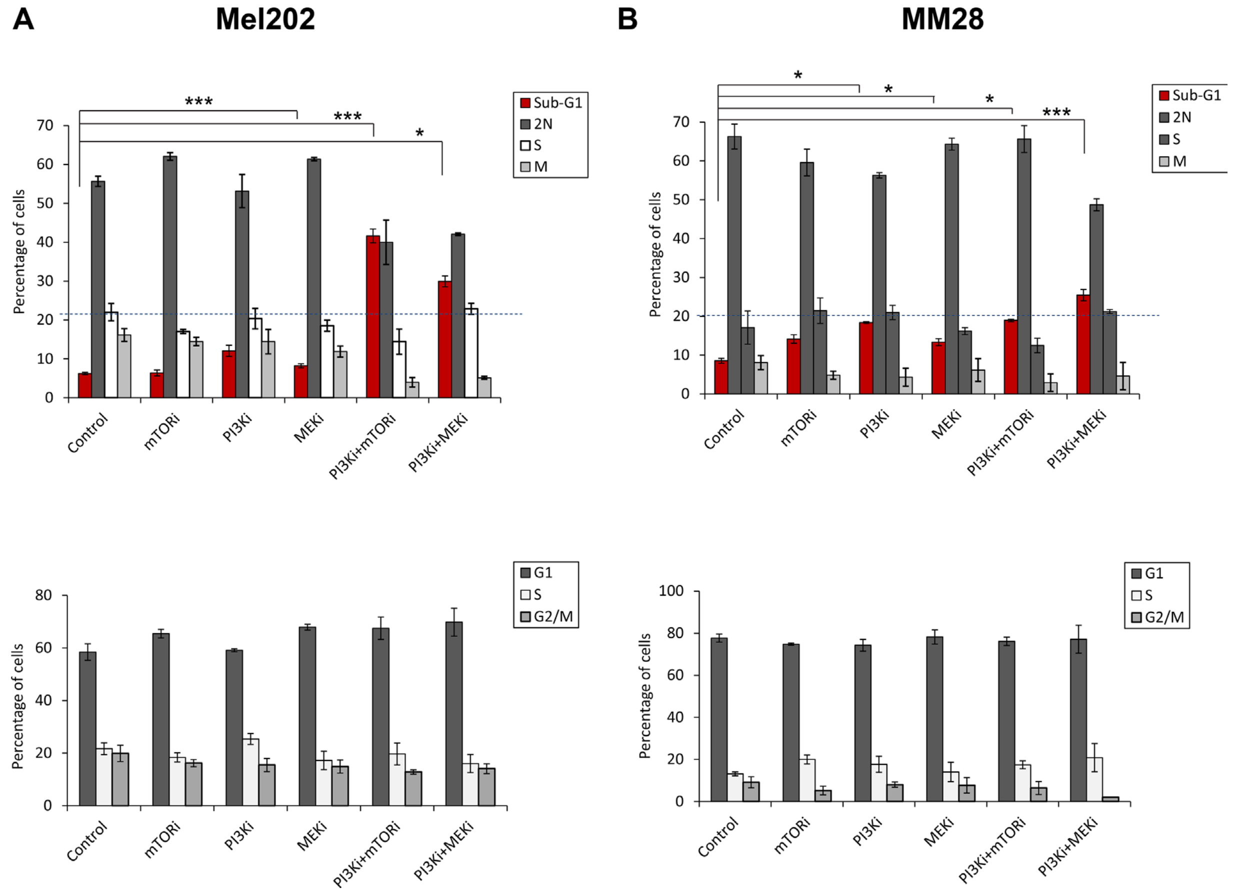

Supplementary Figure S5: Quantification of cell cycle analyses in Mel202 (A) and MM28 (B) cell lines. *Top:* quantification of cell populations in sub-G1, cells with 2N, in S or M phases. *Bottom:* quantification of cell populations in G1, S and G2/M phases. Data are represented as mean ±SEM. Statistical differences between control and treatments in the sub-G1 population was done using two-way ANOVA with with Bonferroni correction. * $p<0.05$; *** $p<0.001$.

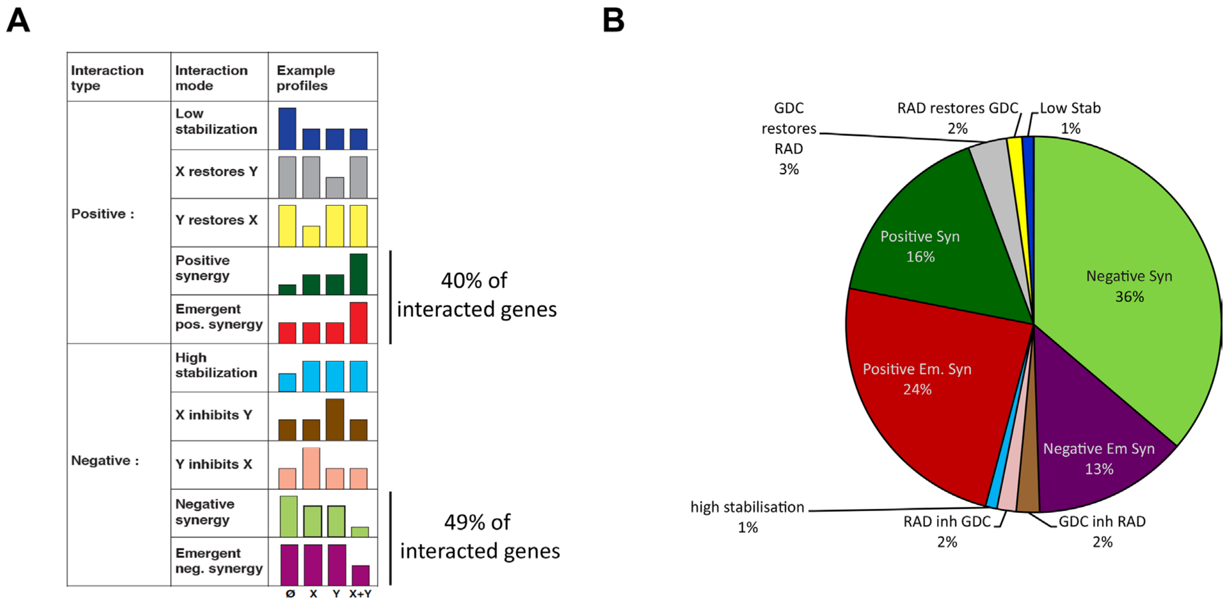

Supplementary Figure S6: Transcriptomic analyses of the Mel202 cell line after dual treatment with PI3K and mTOR inhibitors. A. Table summarizing the different profiles that were used to classified data obtained after transcriptomic analyses. Taken from (1). **B.** Pie chart of the different profiles obtained in the combination treatment at 24h. Percentages depict the proportion of genes in each category.

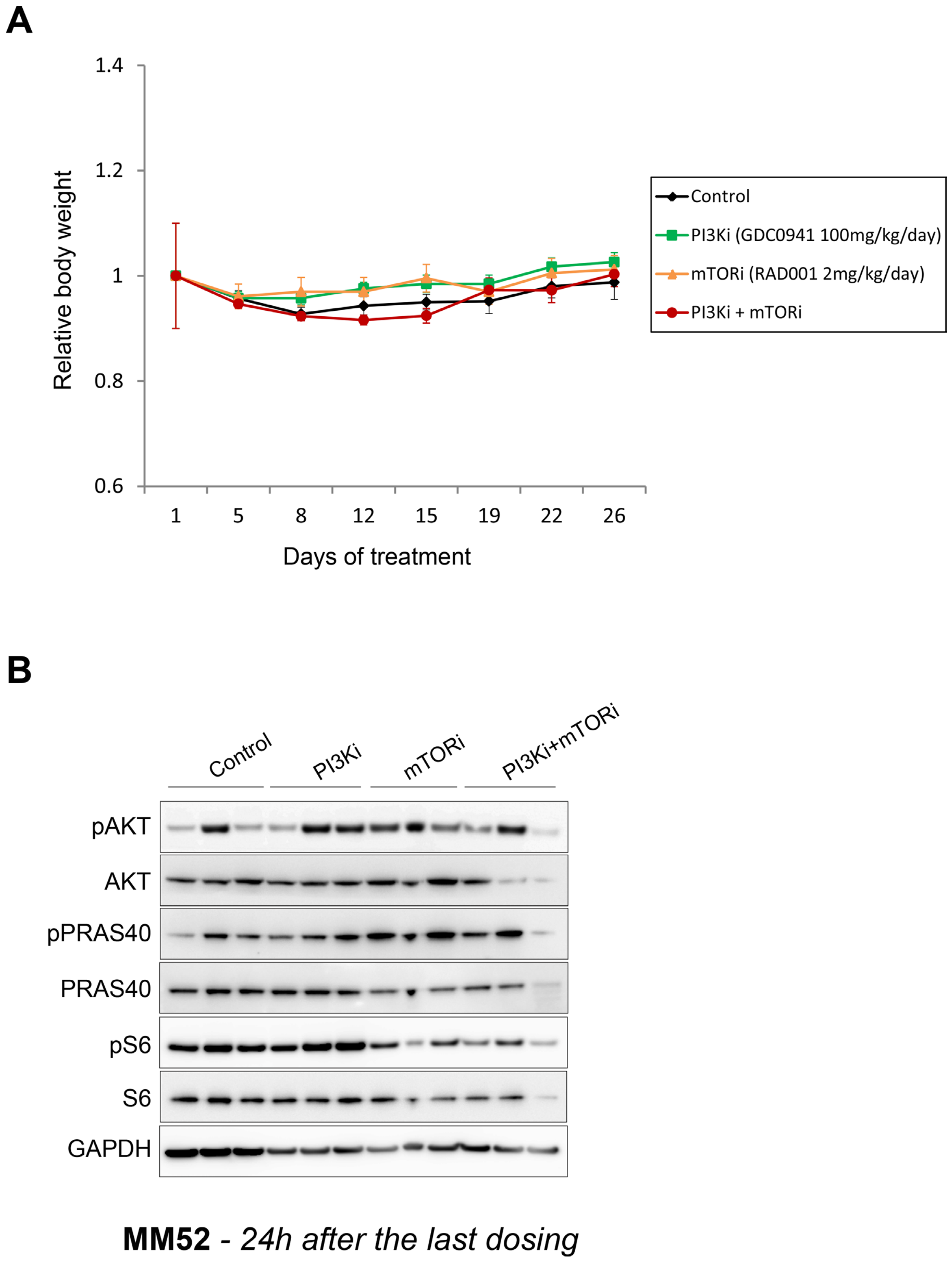

Supplementary Figure S7: *In vivo* evaluation of the PI3K + mTOR inhibitors combination. A. Toxicity assessment in SCID mice. The average relative body weights are shown. Each body weight measure has been normalized to the weight of the corresponding mouse at the beginning of treatment. **B.** Western Blot analyses in the MM52 PDX model at the end of experiment. Samples were collected 24h after the last dosing.
