# Supplementary material for: The mTOR inhibitor Everolimus synergizes with the PI3K inhibitor GDC0941 to enhance anti-tumor efficacy in uveal melanoma
Source: Oncotarget. 2016 Mar 14;7(17):23633–46. doi: 10.18632/oncotarget.8054 (PMC5029652; doi:10.18632/oncotarget.8054)
Supplement: Supplementary file 2 [file oncotarget-07-23633-s002.doc]

**Supplementary Table S3: Gene set enrichment analysis performed on transcriptomic data with David database.**

*Below is a screen snap-shot of the results. More details are presented in the corresponding excel file.*

**
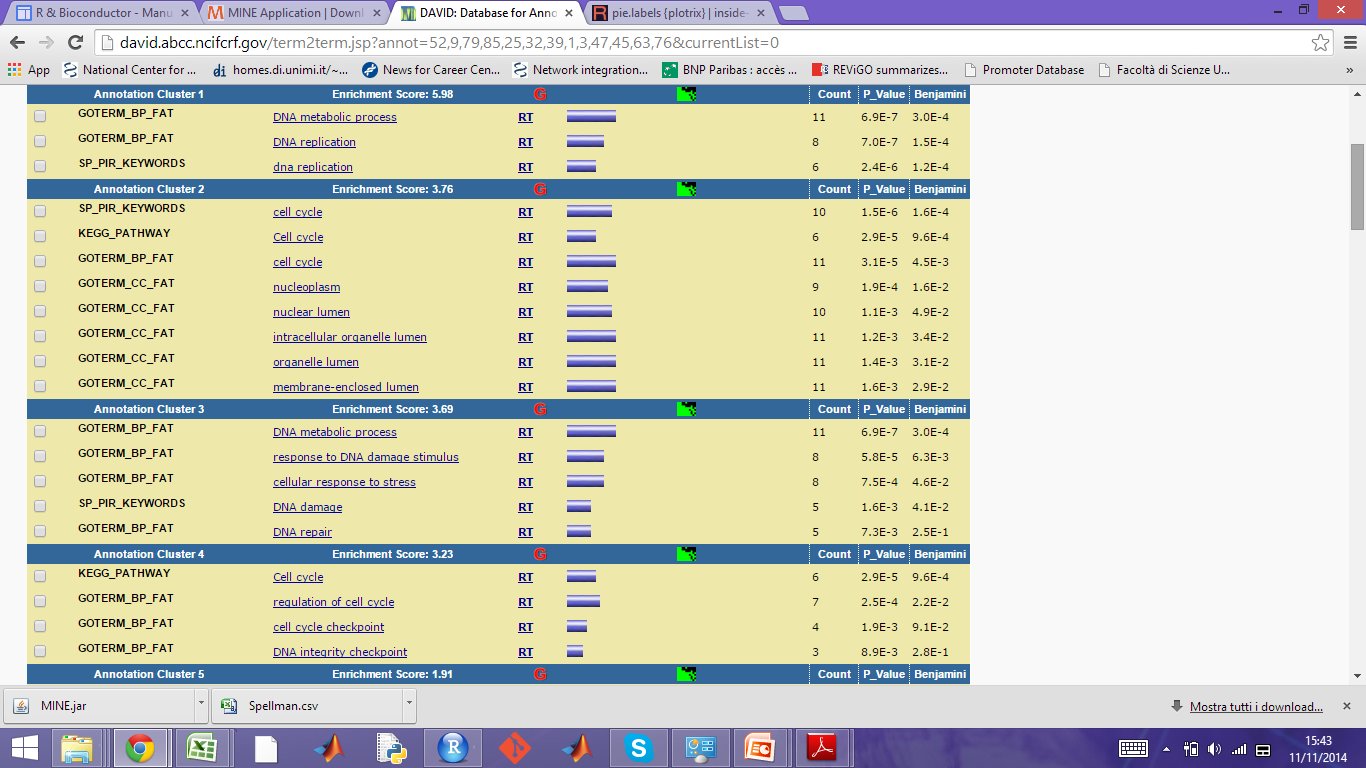
**
